# Explaining the eventual transient saturation of climate-carbon cycle feedback

**DOI:** 10.1186/1750-0680-3-4

**Published:** 2008-04-28

**Authors:** Igor I Mokhov, Alexey V Eliseev

**Affiliations:** 1 A.M. Obukhov Institute of Atmospheric Physics RAS, Moscow, Russia

## Abstract

**Background:**

Coupled climate-carbon cycle simulations generally show that climate feedbacks amplify the buildup of CO_2 _under respective anthropogenic emission. The effect of climate-carbon cycle feedback is characterised by the feedback gain: the relative increase in CO_2 _increment as compared to uncoupled simulations. According to the results of the recent Coupled Climate-Carbon Cycle Model Intercomparison Project (C^4^MIP), the gain is expected to increase during the 21st century. This conclusion is not supported by the climate model developed at the A.M. Obukhov Institute of Atmospheric Physics at the Russian Academy of Sciences (IAP RAS CM). The latter model shows an eventual transient saturation of the feedback gain. This saturation is manifested in a change of climate-carbon cycle feedback gain which grows initially, attains a maximum, and then decreases, eventually tending to unity.

**Results:**

Numerical experiments with the IAP RAS CM as well as an analysis of the conceptual framework demonstrate that this eventual transient saturation results from the fact that transient climate sensitivity decreases with time.

**Conclusion:**

One may conclude that the eventual transient saturation of the climate-carbon cycle feedback is a fundamental property of the coupled climate-carbon system that manifests itself on a relevant time scale.

## Background

Starting from the works [1,2], climate-carbon cycle interactions in the global numerical models attain a lot of scientific attention. These and subsequent works [3-12] have found that an interactive coupling between climate and carbon cycle enhances the build up of the carbon dioxide in the atmosphere, *C*, in comparison to the hypothetical case when the carbon cycle does not respond to the climate changes. If the changes of the atmospheric carbon dioxide in these two cases are $\Delta q_{{\text{CO}}_{2}}^{cpl}$ and $\Delta q_{{\text{CO}}_{2}}^{ucpl}$, respectively, then the parameter of the climate-carbon cycle feedback is

(1) $$f=\Delta q_{{\text{CO}}_{2}}^{cpl}/\Delta q_{{\text{CO}}_{2}}^{ucpl}.$$

In [5], the following expression for climate-carbon cycle feedback gain *g *= (*f *- 1)/*f *is derived

(2) *g *= -*α *(*γ*_*l *_+ *γ*_*oc*_)/(*c*_0 _+ *β*_*l *_+ *β*_*oc*_) = -*αG*

with transient temperature sensitivity $\alpha =dT_{g}^{cpl}/dq_{{\text{CO}}_{2}cpl},T_{g}^{cpl}$ is globally and annually averaged surface air temperature in the coupled simulation, and *c*_0 _= 2.1 *PgC/ppmv*. Coefficients *β*_*X *_and *γ*_*X *_come from linear decomposition of respective differential (i.e., per year) terrestrial and oceanic differential carbon uptakes (*X *= *l *or *X *= *oc *respectively):

$$F_{X}=\beta_{X}\frac{dq_{{\text{CO}}_{2}}}{dt}+\gamma_{X}\frac{dT_{g}}{dt},$$

where *t *stands for time.

For temporal variations of the parameter *f *in the course of a given emission scenario, [13] demonstrated a monotonic increase of *f *during the long-term integration of the Lawrence Livermore National Laboratory coupled model. In the framework of the Coupled Climate-Carbon Cycle Model Intercomparison Project (C^4^MIP) [14], time variations of the respective gains *g *have been studied for the 21st sentury (their Fig. 2b). In the latter case, a tendency for increase of this gain during the course of integration forced by the emission scenario SRES A2 has been exhibited as well. However, according to Fig. 2b in [14], one model exhibits decrease of *g *in the late 21st century and a few others show a slower decrease in the second part of this century in comparison to the first part.

**Figure 1:**
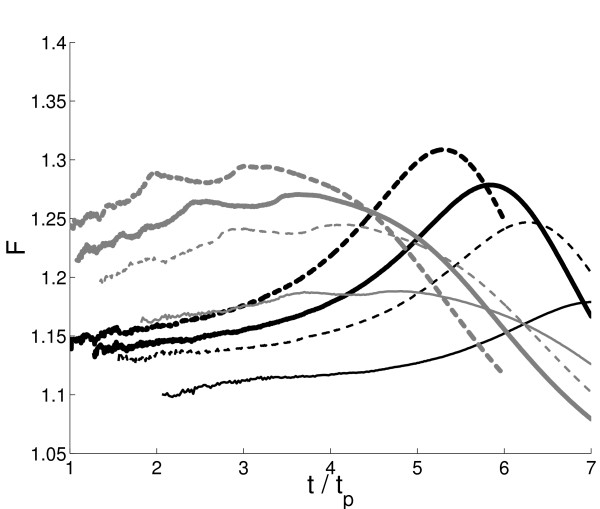
**Parameter of climate-carbon cycle interaction as a function of *t*/*t*_*p*_, obtained in simulations with the IAP RAS CM versions FocL and FocNL (gray and black curves correspondingly) for *t*_*p *_= 50 *yr *(thin solid lines), *t*_*p *_= 100 *yr *(thin dashed lines), *t*_*p *_= 150 *yr *(thick solid lines), and *t*_*p *_= 250 *yr *(thick dashed lines). **In this and subsequent Figures, the data beyond *t *= 7 × *t*_*p *_are not shown because they either out of the simulation length or start to show signs of numerical instability. In addition, data with $\Delta q_{{\text{CO}}_{2}}^{ucpl,cpl}$ < 5 *ppmv *are eliminated from plots.

**Figure 2:**
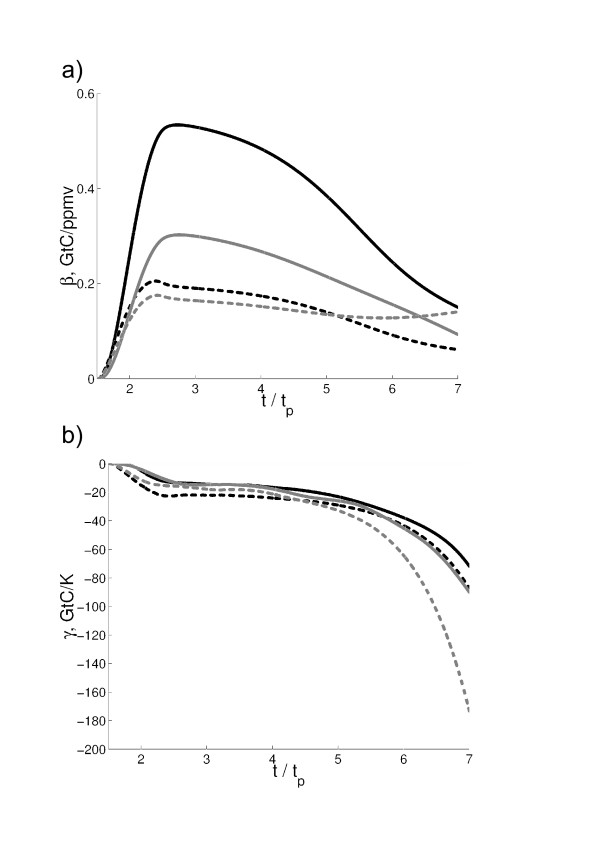
**a) Coefficients *β*_*l *_and *β*_*oc *_(solid and dashed curves respectively) computed for 100-year running segments from the simulations with the IAP RAS CM versions FocL and FocNL (gray and black lines correspondingly) as a function of *t*/*t*_*p*_. **b) Similar to a) but for coefficients *γ*_*l *_and *γ*_*oc*_.

In turn, a non-monotonic change of *f *in the simulation with the IAP RAS climate model of intermediate complexity (IAP RAS CM) under the same scenario SRES A2 was obtained [11,15]. In this simulation, parameter *f *grows during the most part of the run but starts to decrease late in the 21st century. Futher, based on a conceptual climate-carbon cycle model, it has been demonstarted that plausible physical reason for this eventual transient saturation is a weak, logarithmic dependence of the CO_2 _radiative forcing on the atmospheric concentration of carbon dioxide [16]. This leads to small influence of an additional (due to climate-carbon cycle interactions) build up of the carbon dioxide on climate state when *C *is large. Given a length of integration, the latter condition is fulfilled only if emissions are aggressive enough.

However, the build up of the carbon dioxide in the atmosphere in the uncoupled simulation was prescribed in [16] and it is unclear how to relate directly time scales associated with this build up with time scales associated with changes in CO_2 _emissions. In the present paper, ensemble simulations with an Earth system model IAP RAS CM [10-12,15,17] forced by idealised emission scenarios are performed. In this, it is demonstrated that an eventual transient climate-carbon cycle saturation may be exhibited also under moderate emission scenarios if respective integration proceeds for a sufficient time. The results obtained in [16] are supported and it is shown that the above-mentioned hypothesis is proved and even weak but continuing emissions lead to eventual saturation of the climate-carbon cycle feedback. In addition, the IAP RAS CM simulations are supported by integrations with a conceptual coupled model. The latter model is similar, but not identical to that used in [16]. In particular, in the model used here, emissions of CO_2 _are used instead of prescribing the atmospheric carbon dioxide build up in the uncoupled simulation. It is also demonstrated that eventual transient saturation of climate-carbon cycle feedback in the IAP RAS CM is consistent with that derived from the earlier version of conceptual model.

## Results and Discussion

### Analysis of a numerical coupled model

A series of numerical experiments with two IAP RAS CM versions were performed. In one version, oceanic uptake of anthropogenic carbon is formulated as a bilinear function of time derivatives of atmospheric concentration of CO_2 _and globally averaged annual mean sea surface temperature. Hereafter, this version is denoted as FocL. In other version, oceanic uptake of carbon *F*_*oc *_is determined employing a nonlinear model [18] but with chemical constants computed as functions of temperature in accordance to [19]. In particular, this version takes into account carbonate dissolution in the ocean. Hereafter, this version is denoted as FocNL. Terrestrial uptakes for both model versions are determined based on a zero-dimensional module taking into account direct plants fertilisation by CO_2 _and influence of climate (expressed via temperature anomaly from the model's preindustrial state employing *Q*_10_-relationships) on biospheric productivity and autotrophic and heterotrophic respiration. Half-saturation point for the direct plant fertilisation was set to 150 *ppmv *in the version FocL and to 460 *ppmv *in the FocNL. Performance and extensive comparison of these two model versions between each other and with observational estimates are described in [10-12,15,17]. There, it is shown that both versions behave realistically in the 20th century and their projections for the 21st century are basically in line with those obtained in the C^4^MIP project. The simulations performed here were forced by idealised emission scenarios. In these scenarios, fossil fuel emissions increase exponentially in time with a time scale *t*_*p *_from 25 *yr *to 250 *yr *starting from the small initial value *E*_0 _= 0.1 *PgC/yr*. These emissions are not meant to represent any historical or future projected emissions. However, one notes that the combined fossil fuel emissions taken as historical for the 19–20th centiries [20] and adopted from the Special Report on Emission Scenarios [21] for the 21st century (except the scenario B1 where fossil fuel emissions decline in the late 21st century) may be approximated by an exponential curve with *t*_*p *_changing from 50 *yr *to 200 *yr *depending on scenario. One notes that an emission intensity depends both on *E*_0 _and *t*_*p*_. Thus, strictly speaking, quantitative results presented below are only valid for this particular choice of *E*_0_. However, qualitative conclusions are unlikely to be changed if other sufficiently small value of *E*_0 _is selected. In some sense, larger *t*_*p *_may be partly compensated by larger *E*_0_. In the performed simulations, land use emissions are neglected. Every simulation starts from the model's preindustrial equilibrated state and proceeds for 1, 500 *yr*.

As shown in Fig. 1, climate-carbon cycle feedback parameter attains maximum and than eventualy falls down tending to unity. This behaviour is in general agreement with those obtained in the earlier IAP RAS CM simulations [11,15] and employing a conceptual climate-carbon cycle model [16]. There is a difference in timing *t*_*m *_when *f *is at maximum between the two employed here IAP RAS CM versions. For the studied here range of *t*_*p*_, *t*_*m *_ranges from 3 × *t*_*p *_to 5 × *t*_*p *_in simulations with the FocL and from 5 × *t*_*p *_to 8 × *t*_*p *_in simulations with the FocNL. For both model versions, the larger *t*_*p *_the earlier (in units of *t*_*p*_) this maximum of *f *occurs. Difference in responses of the globally and annually averaged surface air temperatures between coupled and uncoupled simulation also increases early in the simulations, then attains a maximum (which occurs later than *t*_*m*_) and diminishes afterwards tending to zero (not shown). To diagnose the behavior of the model, an approach by [5] has been adopted. In this, coefficients *γ*_*X *_and *β*_*X *_(*X *= *l, oc*) entering (2) were computed for the running 100-yr segments. For both model versions, *β*_*l *_increases till the plants fertilisation half-saturation point is reached and then decreases to zero (see Fig. 2a as an example). This behaviour reflects the Michaelis-Menten-type dependence of terrestrial productivity on $q_{{\text{CO}}_{2}}$ as it is implemented in the IAP RAS CM. Coefficient *β*_*oc *_behaves similarly in the version FocNL while changes in time only slightly in the version FocL. This is expected from the formulations of *F*_*oc *_in these model versions. In contrast, the magnitudes of *γ*_*l *_and *γ*_*oc *_increse during the course of integration (Fig. 2b).

**Figure 3:**
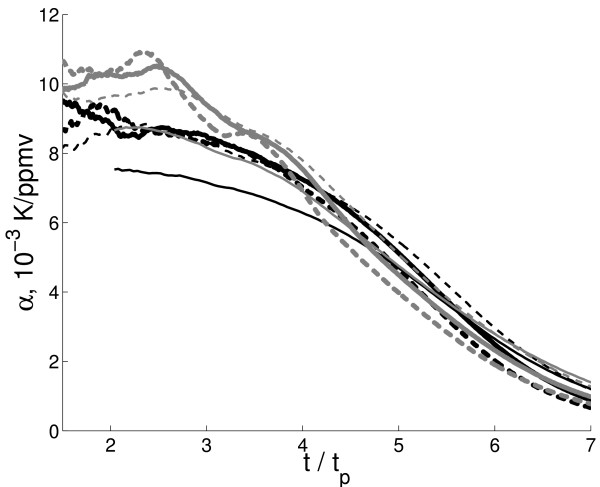
**Transient temperature sensitivity *a *computed for 100-year running segments from the simulations with the IAP RAS CM versions FocL and FocNL with different *t*_*p*_.** Notations are the same as in Fig. 1.

This behaviour of *β*s and *γ*s obviously leads to the magnitude of *G *(see (2)) increasing with time if the latter is large enough (perticularly, after *t*_*m*_, not shown). Consequently, general decrease of gain *g *and feedback parameter *f *is due to drastic decrease of transient temperature sensitivity *α *in the course of integration. Being computed for 100-yr running segments, *α *shows almost monotonic decrease during the course of integration by an order of magnitude (Fig. 3). This decrease overcompensates an increase of *G *with time. Such a decrease of sensitivity is again consistent with a commonly accepted weak, logarithmic dependence of the carbon dioxide instantaneous radiative forcing on the CO_2 _atmospheric content. To get an impression, how large $q_{{\text{CO}}_{2}}$ should be for eventual transient saturation, the time when *f *eventually becomes smaller than 1.05 is computed and the corresponding value $q_{{\text{CO}}_{2},s}$ is stored. This value ranges between 3226 *ppmv *and 6152 *ppmv *(3679 *ppmv *and 12799 *ppmv*) for the version FocL (FocNL). The smaller values in these ranges correspond to the larger values of *t*_*p*_. While the upper part of these ranges seems unrealistic (especially that obtained for the version FocNL), the lower part can be achieved if future CO_2 _emission come along the worst (e.g., SRES A2) path. It is important with this respect that this lower part of range is obtained for *t*_*p *_= 150 – 250 *yr *which is applicable for the SRES scenarios.

### Analysis of a conceptual model

In this section, a conceptual coupled model similar, but not identical to [16] is employed. An important distinction between the present paper and [16] is as follows. In [16], a build up of carbon dioxide in the atmosphere in the uncoupled simulation is prescribed. In contrast, in the present paper, the model is forced by idealised emission scenarios. This results in different time scales used in these two papers. Here, *t*_*p *_means time scale of emission growth while in the earlier paper the time scale for $q_{{\text{CO}}_{2}}^{ucpl}$ growth was used. The latter approach implicitly includes all details of emissions and direct fertilisation via prescribed curve $q_{{\text{CO}}_{2}}^{ucpl}(t)$. However, the time scale associated with $q_{{\text{CO}}_{2}}^{ucpl}(t)$ is larger than *t*_*p *_by an order of magnitude. As a result, to be consistent with the above analysis of the IAP RAS CM, an emission time scale is used here. The equations for the airborne carbon storage for the coupled and uncoupled cases are, respectively,

(3) $$c_{0}dq_{{\text{CO}}_{2}}^{cpl}/dt=E-F_{oc}^{cpl}-F_{l}^{cpl},$$

(4) $$c_{0}dq_{{\text{CO}}_{2}}^{ucpl}/dt=E-F_{oc}^{ucpl}-F_{l}^{ucpl},$$

where *c*_0 _= 2.1 *PgC/ppmv*, *E *stands for emissions, and *F*_*l *_and *F*_*oc *_are terrestrial and oceanic carbon uptakes, respectively. Linearising carbon fluxes [5] and neglecting difference between the changes of globally averaged annual mean surface air and sea surface temperatures one obtains

(5) $$\begin{array}{l} F_{X}^{cpl}=\beta_{X}dq_{{\text{CO}}_{2}}^{cpl}/dt+\gamma_{X}dT_{g}^{cpl}/dt,\\ F_{X}^{ucpl}=\beta_{X}dq_{{\text{CO}}_{2}}^{ucpl}/dt, \end{array}$$

where *X *= *l, oc*, *T*_*g *_stands for globally averaged annual mean temperature, and *γ*_*X *_and *β*_*X *_were defined in Introduction. From (3)–(5) one derives

(6) $$\Delta q_{{\text{CO}}_{2}}^{cpl}=\Delta q_{{\text{CO}}_{2}}^{ucpl}+\kappa \Delta T_{g}^{cpl},$$

with *κ *= -*G *= (*γ*_*l *_+ *γ*_*oc*_)/(*c*_0 _+ *β*_*l *_+ *β*_*oc*_), see Eq. (2). For the C^4^MIP models under the emission scenario SRES A2, from [14] one derives *κ *= (8 – 48) *ppmv/K *for the 21st century. Coefficient *β *= *β*_*l *_+ *β*_*oc *_varies from 1.7 *PgC/ppmv *to 3.7 *PgC/ppmv *with a mean value 2.5 *PgC/ppmv *[14]. For the the IAP RAS CM ensemble simulation, *κ *depends on *t*_*p*_. Here, at a time short before *t*_*m*_, *κ *varies from very small values for *t*_*p *_= 25 *yr *up to 25 *ppmv/K *(35 *ppmv/K*) for the version FocNL (FocL) if *t*_*p *_= 250 *yr*. Coefficient *β *depends on *t*_*p *_as well. At time of a few *t*_*p*_, it again attains very small values for *t*_*p *_= 25 *yr *and amounts 1.6 *PgC/ppmv *(2.5 *PgC/ppmv*) for the version FocL (FocNL) for *t*_*p *_= 250 *yr*.

For the same exponentially growing emissions as those used to force the IAP RAS CM in the previos Subsection, and for the uncoupled case, Eq. 4 may be integrated analytically to give

(7) $$q_{{\text{CO}}_{2}}^{ucpl}=q_{{\text{CO}}_{2},0}+\frac{E_{0}t_{p}}{c_{0}+\beta }(\exp(t/t_{p})-1)$$

with the initial atmospheric carbon dioxide concentration *C*_0_.

For coupled simulation, one may write [22]

(8) $$F_{H}^{cpl}=R^{cpl}-\lambda \Delta T_{g}^{cpl}$$

where $F_{H}^{cpl}$ is oceanic heat uptake, *R*^*cpl*^is radiative forcing. The latter is substituted as $R^{cpl}=R_{0}\ln\left(q_{{\text{CO}}_{2}}^{cpl}/q_{{\text{CO}}_{2},0}\right)$ with *R*_0 _= 5.4 *Wm*^-2^*K*^-1 ^[21,23]. Climate feedback parameter is $\lambda =R_{0}\ln2/\Delta T_{2\times {\text{CO}}_{2}}$ with $\Delta T_{2\times {\text{CO}}_{2}}$ standing for the equilibrium model sensitivity to doubling of the atmospheric carbon dioxide concentartion. Upon substituting $F_{H}^{cpl}=Cd\Delta T_{g}^{cpl}dt$ (*C *is heat capacity per unit area) one gets

(9) $$C=\frac{d\Delta T_{g}^{cpl}}{dt}=R_{0}\ln\left(1+\frac{\Delta q_{CO_{2}}^{cpl}}{q_{CO_{2},0}}\right)-\lambda \Delta T_{g}^{cpl},$$

Alternatively, Eq. (9) may be treated as a simple zero-dimensional climate model [24].

Eqs. (6), (7) and (9) have been numerically integrated subject to the above-mentioned exponential emissions (expressed via (7)) and to initial condition ${\Delta T_{g}^{cpl}|}_{t=0}=0$. An ensemble of conceptual model integrations is performed varying *t*_*p *_in the same range as it was for IAP RAS CM and varying *β *in the range from 0 to 5.0 *PgC/ppmv *(this range is wider than the corresponding C^4^MIP range, see above). Equilibrium model sensitivity to doubling of the carbon dioxide in the atmosphere was varied between 1 *K *and 9 *K *roughly corresponding to combined range from [25-28] (in [29], a narrower range from 2.0 *K *to 4.5 *K *is figured). Parameter *κ *was varied from 8 *ppmv/K *to 48 *ppmv/K *(see above).

**Figure 4:**
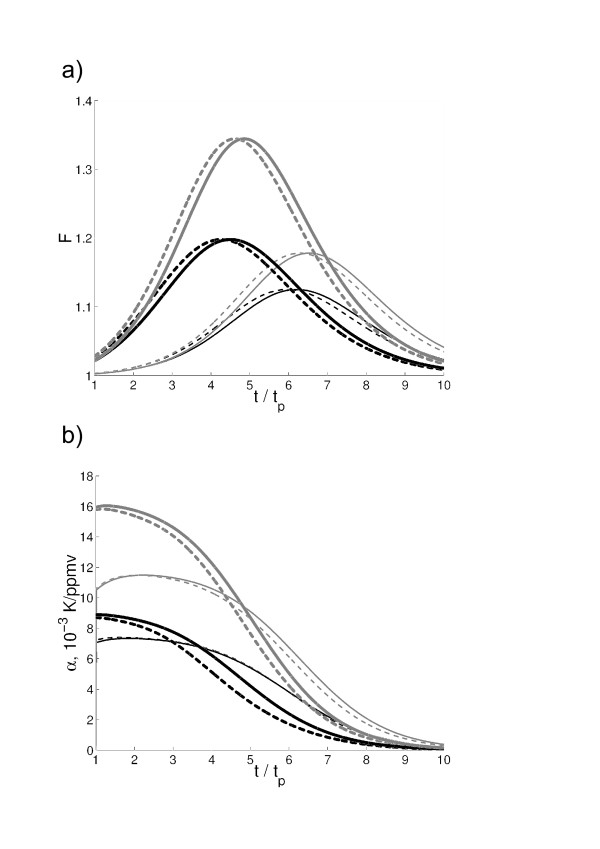
**Parameter of climate-carbon cycle interaction in the conceptual model (a) and transient temperature sensitivity *α *computed for 100-year running segments (b) as functions of *t*/*t*_*p*_.** Shown are curves for *t*_*p *_= 50 and 150 *yr *(thin and thick lines respectively) with *β *= 1.5 and 2.5 *PgC/ppmv *(dashed and solid lines correspondingly) and $\Delta T_{2\times {\text{CO}}_{2}}$ = 2 and 4 *K *(black and gray lines respectively) for *κ *= 28 *ppmv/K*.

In all these integrations, *f *attains maximum and then falls down to unity (Fig. 4a). The timing of this maximum is *t*_*m *_= (2 – 8) × *t*_*p *_depending on governing parameters. The latter range is similar to that obtained in the simulations with the IAP RAS CM despite of the fixed value of $\Delta T_{2\times {\text{CO}}_{2}}$ of smaller range of variations in *β *in the IAP RAS CM. In particular, the latter parameter was varied only between two values, one corresponding to the version FocL and the other corresponding to the version FocNL. Moreover, if a narrower range of $\Delta T_{2\times {\text{CO}}_{2}}$ figured in [29] is considered and/or *β *is constrained to be in a C^4^MIP range, an interval for *t*_*m*_/*t*_*p *_shrinks only slightly in the integrations with a conceptual model. The obtained *t*_*m*_/*t*_*p *_is insensitive to variations in *κ *This is consistent with the results obtained in [16]. Typically, *t*_*m*_/*t*_*p *_increases if any of $\Delta T_{2\times {\text{CO}}_{2}}$ and *β *increases or *t*_*p *_decreases (see Fig. 4a). For variations in *t*_*p*_, this behaviour is consistent with that obtained employing the IAP RAS CM. For variations in *β*, it is consistent as well since combined *β *for the version FocL is larger than for the version FocNL (basically, due to larger *β*_*oc *_for the former model version, see [15]).

In a conceptual model, by construction, *G *= -*κ *does not change during the course of integration. As a result, as it was for the IAP RAS CM, eventual tending of *f *to unity is due to decrease in transient temperature sensitivity *a *during the course of integration (Fig. 4b). In turn, this is due to weak, logarithmic dependence of the CO_2 _radiative forcing on the atmospheric concentration of carbon dioxide. One may estimate $q_{{\text{CO}}_{2},s}$ from this conceptual model as well. For simplicity, one may assume that temperature response to carbon dioxide forcing is stationary. This is achieved by dropping $F_{H}^{cpl}$ in Eq. (8). Taking into accout Eq. (6), one obtains

(10) $$\frac{\kappa \Delta T_{2\times {\text{CO}}_{2}}}{q_{{\text{CO}}_{2},s}\ln2}\ln\frac{q_{{\text{CO}}_{2},s}}{q_{{\text{CO}}_{2},0}}=0.05$$

where again the threshold value for *f *is set to 1.05. This equation has been solved numerically appling the dichotomy method. In general, the larger *κ *and/or $\Delta T_{2\times {\text{CO}}_{2}}$ the larger $q_{{\text{CO}}_{2},s}$. For $\Delta T_{2\times {\text{CO}}_{2}}$ = 2.2 *K*, the values obtained for the conceptual model are similar, but slightly larger, to those estimated from the IAP RAS CM runs.

As an aside issue, one may quantify how "small" *E*_0 _should be in order not to make the approach followed here meaningless. Physically, *E*_0 _has not to result in sudden kink in $q_{{\text{CO}}_{2}}^{ucpl}$ at *t *= 0. From Eq. (7), it needs

(11) $$E_{0}\ll (c_{0}+\beta )q_{{\text{CO}}_{2},0}/t_{p}\leq c_{0}q_{{\text{CO}}_{2},0}/t_{p}\leq 2.4\;PgC/yr.$$

The value of *E*_0 _selected in this paper, 0.1 *PgC/yr*, fulfills this condition. If (11) is satisfied then qualitative conclusion of the present paper and the respective conclusions related to the order of magnitudes of basic characteristics are expected to remain unchanged.

## Discussion

An eventual transient saturation, as studied in the present framework, implies that initially (at a time ≪ *t*_*p*_) and eventually (at a time ≫ *t*_*p*_), the state of the coupled system is close to the state of the uncoupled one. However, during an intermediate period when *t*' ≃ *t*_*p*_, *f *deviates substantinally from unity and climate-carbon cycle interaction does matter. Taking into account typical time scales of CO_2 _emission growth for the SRES emission, *t*_*p *_= 50 – 200 *yr*, this intermediate period extends for the next several centuries.

While it is beyond the scope of the present paper to concern CO_2 _emissions changing non-exponentially in time, it is possible to make a note with this respect. In particular, if the future emissions would change in time slower than the exponential ones, it would be expressed in apparent increase of *t*_*p *_during the course of simulation. In turn, the timing *t*_*m *_of maximum of *f *and an eventual transient saturation would be delayed in comparison to the case with constant *t*_*p*_. On the other hand, for faster-than-exponential emissions this eventual transient saturation would come closer to the present day.

The long-term fate of anthropogenic carbon is basically governed by calcium carbonate dissolution in the ocean [30,31]. This effect is taken into account only in the version FocNL and neglected in the version FocL and in the conceptual model. However, carbonate dissolution is important only for time scales larger than ≈ 5, 000 *yr *[30] which is well above the length of every individual simulation performed in the present paper (1, 500 *yr*). As a result, the version FocL and the conceptual climate-carbon cycle model are applicable for the problem considered here as well.

Returning to C^4^MIP simulations, one notes that it is possible to find some hints for this eventual transient saturation in these simulations [14] as well (see Background). However, in the C^4^MIP integrations, an eventual climate-carbon cycle carbon saturation is less marked. The most probable reason for this is the length of the C^4^MIP simulations which is too small to make this saturation visible given an emission intensity in these simulations. One expects that if these simulations would extended to the future, eventually, parameter *f *would converge to unity in the C^4^MIP ensemble as well. Some complications for this convergence could arise from a specific behaviour of *a *for particular models, e.g., due to behaviour of the oceanic heat uptake in the course of integration and its interrelation with equilibrium temperature sensitivity [32-35].

As a final note, one may distinguish manifestation of a climate-carbon cycle feedback eventual transient saturation in terms of feedback parameter, on one hand, and in terms of feedback gain, on the other one. In particular, as *dg/dt *= *f*^-2^*df*/*dt *and, generally, *f *> 1 in the course of simulations, this saturation is somewhat masked for *g *in comparison to *f*. This also adds to the masking of the eventual transient saturation of the climate-carbon cycle feedback in the C^4^MIP simulations.

## Conclusion

In this paper, simulations with the IAP RAS climate model of intermediate complexity have been performed to study temporal variations of the climate-carbon cycle feedback parameter. Two model versions were considered differing between each other by the formulation of the oceanic uptake of carbon dioxide and by the governing parameters of the terrestrial carbon uptake module. Both versions were forced by idealised scenarios of fossil fuel emissions. In these scenarios, emissions grow exponentially in time (with a characteristic timescale *t*_*p *_= 25 – 250 *yr*) starting from a small initial emission value. Land use emissions were set to zero. In all simulations with both model versions, climate-carbon cycle feedback parameter *f *grows initially, attains maximum, and then decreases eventually tending to unity. The timing of this maximum is of the order of a few *t*_*p *_for the selected small value of initial emission. This general behaviour is consistent to that obtained in a earlier simulations with the same model.

In particular, coefficients of climate-carbon cycle interaction diagnosed for 100-yr running segments lead to increase of climate-carbon cycle gain *g *during the course of integration. This increase, however, is overcompensated by decrease of transient climate sensitivity *α*. The latter leads to the above-mentioned eventual transient saturation of climate-carbon cycle feedback.

The IAP RAS CM simulations are supported by an analysis of a conceptual model with linear dependence of carbon sinks from the atmosphere on changes in atmospheric carbon dioxide concentration and global temperature rise. This simple model exhibits an eventual transient saturation of the climate-carbon cycle feedback which is similar to the model of intermediate complexity.

## Competing interests

The authors declare that they have no competing interests.

## Authors' contributions

Both authors equally contributed to the paper.
